# Genome-wide association study and scan for signatures of selection point to candidate genes for body temperature maintenance under the cold stress in Siberian cattle populations

**DOI:** 10.1186/s12863-019-0725-0

**Published:** 2019-03-18

**Authors:** Alexander V. Igoshin, Andrey A. Yurchenko, Nadezhda M. Belonogova, Dmitry V. Petrovsky, Ruslan B. Aitnazarov, Vladimir A. Soloshenko, Nikolay S. Yudin, Denis M. Larkin

**Affiliations:** 1 The Federal Research Center Institute of Cytology and Genetics, The Siberian Branch of the Russian Academy of Sciences (ICG SB RAS), 630090 Novosibirsk, Russia; 2 Siberian Research Institute of Animal Husbandry, 630501 Krasnoobsk, Russia; 3 Novosibirsk State University, Novosibirsk, Russia; 4 Royal Veterinary College, University of London, London, NW1 0TU UK

**Keywords:** Body temperature, GWAS, DCMS, Cattle, Selection, Adaptation, Cold climate, *GRIA4*, *MSANTD4*

## Abstract

**Background:**

Design of new highly productive livestock breeds, well-adapted to local climatic conditions is one of the aims of modern agriculture and breeding. The genetics underlying economically important traits in cattle are widely studied, whereas our knowledge of the genetic mechanisms of adaptation to local environments is still scarce. To address this issue for cold climates we used an integrated approach for detecting genomic intervals related to body temperature maintenance under acute cold stress. Our approach combined genome-wide association studies (GWAS) and scans for signatures of selection applied to a cattle population (Hereford and Kazakh Whiteheaded beef breeds) bred in Siberia. We utilized the GGP HD150K DNA chip containing 139,376 single nucleotide polymorphism markers.

**Results:**

We detected a single candidate region on cattle chromosome (BTA)15 overlapping between the GWAS results and the results of scans for selective sweeps. This region contains two genes, *MSANTD4* and *GRIA4*. Both genes are functional candidates to contribute to the cold-stress resistance phenotype, due to their indirect involvement in the cold shock response (*MSANTD4*) and body thermoregulation (*GRIA4*).

**Conclusions:**

Our results point to a novel region on BTA15 which is a candidate region associated with the body temperature maintenance phenotype in Siberian cattle. The results of our research and the follow up studies might be used for the development of cattle breeds better adapted to cold climates of the Russian Federation and other Northern countries with similar climates.

**Electronic supplementary material:**

The online version of this article (10.1186/s12863-019-0725-0) contains supplementary material, which is available to authorized users.

## Background

Adaptation of livestock breeds to local climatic conditions is an important trait for contemporary agriculture because it reduces the environmental stress put on animals, and leads to an increased and more environmentally-friendly production [1]. With over 1000 breeds existing worldwide in various environments e.g., hot and cold climates, cattle are an excellent model to study genetic adaptations. These studies can be performed using different approaches, including: a) scans for signatures of selection within breeds adapted to a specific environment, including genomes of breeds which are adapted to contrasting conditions; b) genome-wide association studies (GWAS) within populations looking for a range of animal reactions to environmental factors; and c) checking for expression and/or variations within or near candidate genes identified using these approaches or reported for other species (comparative genomics). It is promising to combine complementary approaches in a single or consecutive studies when detecting candidate genes for traits that are not under intensive artificial selection for many generations (therefore found in relatively short haplotype blocks compared to regions under intensive selection [2, 3]). An animal’s ability to maintain its body temperature under acute cold stress is an exemplar of such a trait, at least for livestock species, as for decades it was not on the list of breeding programs.

There are studies which attempted to collect information on genes related to cold adaptation in mammals to facilitate the use of comparative genomics approaches [4, 5]. One example is our recent work on building a compendium of genes under positive selection in mammals from the Arctic or Antarctic [6]. While we identified 416 genes which were under positive selection in two species there were only 12 genes shared by three species and none shared by more than three (out of the six species we looked at). This could indicate that adaptation to cold environments is a complex process which often involves different pathways (or different genes within pathways) in different species, suggesting that detailed studies of cold response in livestock (e.g., cattle) are required and we cannot rely only on the data originating from comparative genomics to identify candidate genes.

We recently looked for genomic intervals under putative selection in Russian native breeds of cattle, including several breeds which are adapted to extreme cold environments (i.e. the Yakut cattle which can be found above the Polar Circle and adapted to survive very cold winters (down to -50 °C) [7]. Our work has revealed multiple candidate genes that could be attributed to cold climate adaptation. The strongest candidates were the *AQP5* previously reported as a cold acclimatization candidate because of its role in water channel formation [5] and *RETREG1* known to cause the impairment of pain and temperature sensation in humans [8]. In a complementary study, Yang and colleagues (2017) performed the detection of copy number variations (CNVs) in eight Chinese native cattle breeds and found two CNVs specific to Northern breeds which may play role in adaptation to the cold climate of Northern China. These CNVs contained genes: *TMC6,* which could affect milk quality in cold environment, and *COL27A1,* which has been proposed to play role in cartilage calcification which is important in a cold climate [9].

Another group of studies investigated the reaction of animals and changes in gene expression in response to a short period of cold stress or in response to seasonal climate changes. Howard and co-workers (2014) performed a GWAS for body temperature regulation in cattle during a five-day period of heat and cold stresses. The study on reaction to cold stress has identified several candidate genes involved in: energy metabolism (*COX7C*), vasculogenesis (*RASA1*), pentose phosphate pathway (*FBP1*, *FBP2*), heat shock protein response (*HSBP1*), ion regulation (*PRKCB*, *CACNG3*), and thyroid hormone regulation (*TRIP11*) [10]. In addition, Xu and colleagues (2017) conducted a global gene expression profiling from the blood of cattle exposed to acute cold stress. They revealed a total of 193 genes which alter expression profile after a three-hour cold (− 32 °C) exposure [11]. Thirteen biological networks were affected, including cellular compromise, cellular movement, lipid metabolism, molecular transport, cell death, and survival. Pawar and colleagues (2014), using a set of candidate genes previously identified as candidates for climate adaptations, demonstrated changes in the expression level of heat shock genes (*HSF1, HSP70*), interleukin (*IL-12*), tumour necrosis factor-alpha (*TNF*-alpha), and the granulocyte macrophage colony stimulating factor (*GMCSF*) in blood of buffaloes between the winter and summer seasons [12]. A similar study by Kumar and colleagues (2015) explored seasonal variations in the expression level of three *HSP70* family genes, as well as *HSP10*, *HSP60*, *HSP90,* and *HSF1* in Sahiwal and Tharparkar breeds of zebu cattle (*Bos indicus*), and the Murrah buffalo (*Bubalus bubalis*) in India. Most of the genes demonstrated upregulation during the winter and summer seasons in comparison to spring [13].

As it follows from the examples above, there is little (if any) overlap between genes within the same species (cattle) which are detected in different studies of selection in response to adaption to cold climates, and the reaction of an organism to a short period of cold exposure and seasonal changes of temperature. This implies that combining several approaches in a single study to detect genes affected in an organism’s response to cold, if successful, could be a promising way of finding such genes for a specific environment and for a specific breed or group of breeds. To test this hypothesis, we performed a GWAS for the ability to maintain body temperature during exposure to extreme cold in a population of two related breeds from Siberia: Hereford and the Kazakh Whiteheaded. Both breeds were bred in Siberia for several decades, therefore expected to be adapted to the local environment. On the other hand, these breeds were originally established in areas with a moderate climate, implying that segregation of cold adaptation genotypes could still be present within the population. These data were combined with the results of scans for signatures of selection within the same population, which could point to genome intervals affected during the breed formation, artificial selection, and acclimation. The results indicate that our integrative approach was useful in detecting novel candidate genes involved in the response of an organism to acute cold.

## Results

### Filtering and population structure

The principal component analysis (PCA) has demonstrated that the Hereford and Kazakh Whiteheaded individuals form two separate clusters. However, when using the first two components (PC1, PC2) one Hereford individual clustered with animals of the Kazakh Whiteheaded breed while three individuals of the Kazakh Whiteheaded breed were found within the Hereford cluster (see Additional file 1: Figure S1). Breed identifiers were reassigned for all these animals to match clustering results (see Additional file 2). Genotyping quality controls removed 14,364 single nucleotide polymorphisms (SNPs) not assigned to a specific chromosome or position within the chromosome and 5171 SNPs found on sex chromosomes, resulting in 119,841 SNPs used in the haplotype and linkage disequilibrium (LD) block inference. For the SNP-based GWAS studies and signature of selection analysis, an additional 11,685 SNPs were removed due to low minor allele frequency (MAF), deviation from the Hardy-Weinberg equilibrium and low call rate for our samples resulting in 108,156 SNPs used in these analyses.

### SNP-based genome-wide association study

**Fig. 1:**
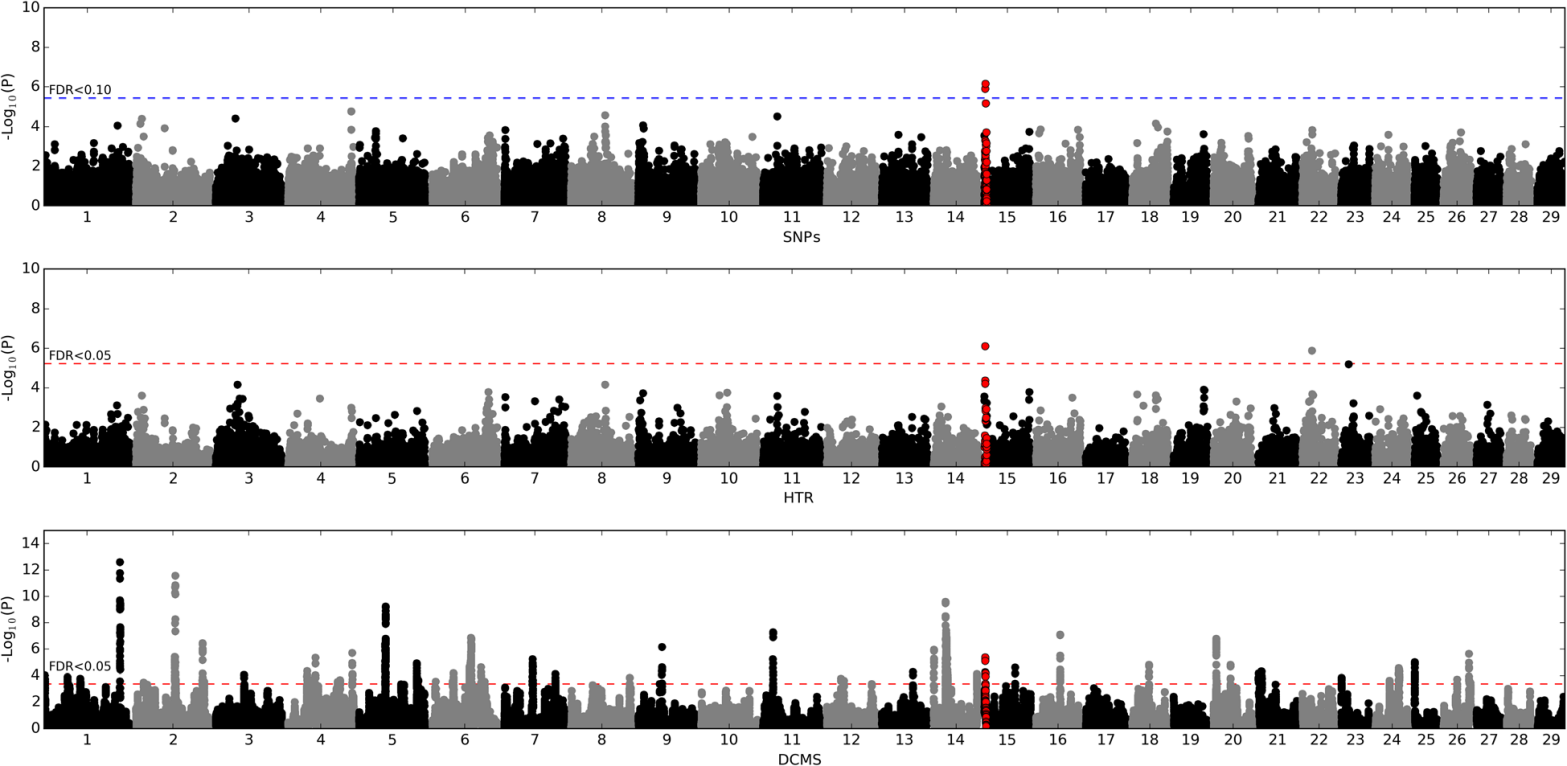
Manhattan plots for the SNP- and haplotype-based GWAS and DCMS analyses. In red, the region on BTA15 overlapping between all types of analyses is shown. Dotted horizontal lines indicate FDR thresholds at suggestive (FDR < 0.1) level (in blue) and significant (FDR < 0.05) level (in red)

**Fig. 2:**
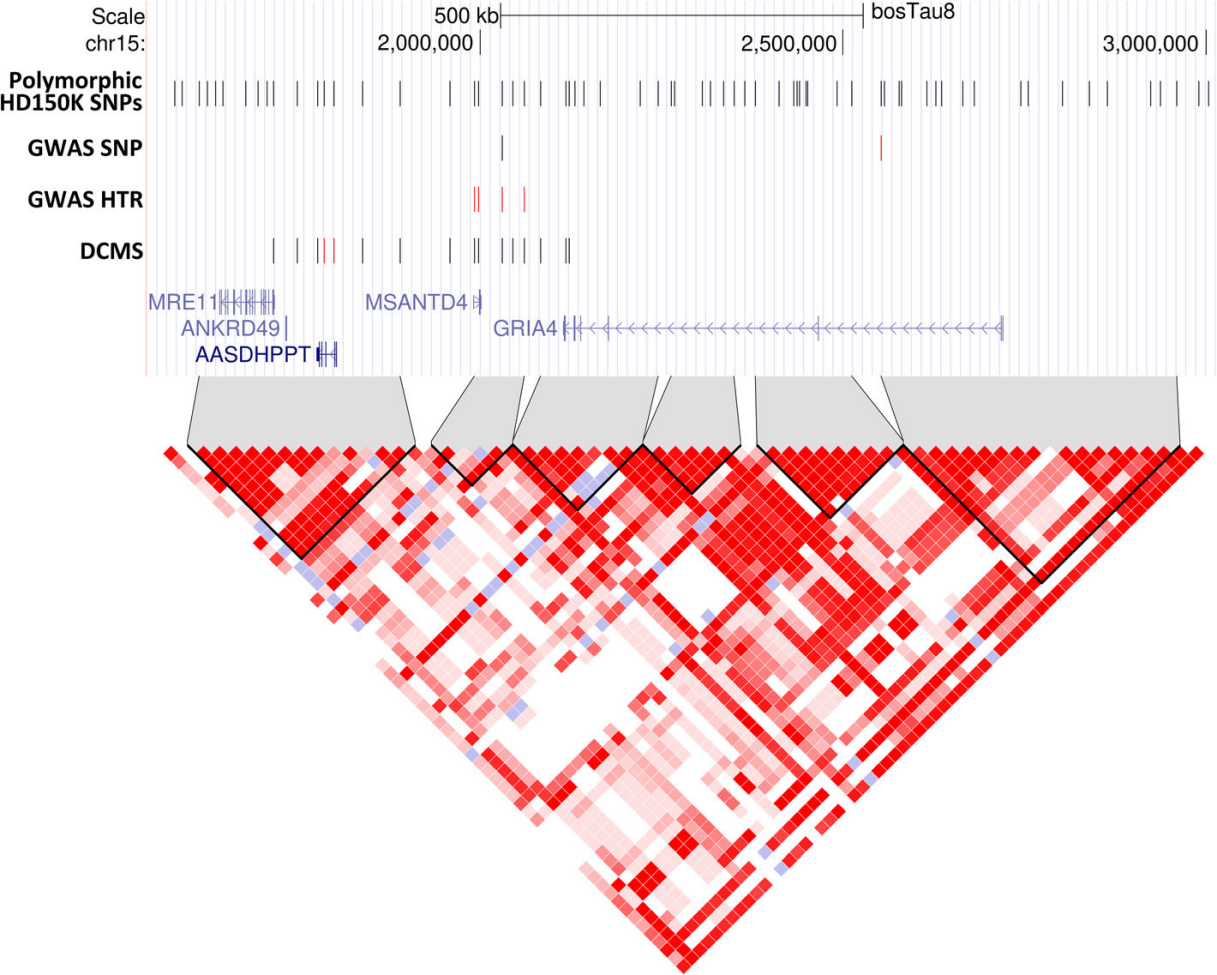
SNPs, genes and haploblock structure of the region on BTA15 indicated by three types of analysis. For each type of analysis vertical lines represent SNPs which passed the FDR threshold (FDR < 0.1 for SNP-based GWAS and FDR < 0.05 for haplotype-based GWAS and DCMS). Red vertical lines indicate SNPs with the lowest q-values. The red triangle represents haploblock structure of the region with grey shading areas connecting haploblocks to the corresponding chromosome intervals

In the GWAS, breed, sex, relatedness, and population stratification were accounted for in our EMMAX model. The genomic inflation factor was 0.98 suggesting that most population bias was addressed; therefore no additional corrections were applied (see Additional file 1: Figure S2). The analysis has revealed two SNPs located on cattle chromosome (BTA) 15 at the suggestive level of significance (Fig. 1; Table 1). The most significant SNP was the BovineHD1500000589 (*p*-value = 9.55 × 10^− 7^, q-value = 0.068) found within an intron of the *GRIA4* gene (Fig. 2 and Table 1). The second SNP was the BovineHD1500000472 (*p*-value = 1.7 × 10^− 6^, q-value = 0.068), located on BTA15 between the *MSANTD4* and *GRIA4* genes. The two SNPs were found to be in LD (r^2^ = 0.82 and D’ = 1).

**Table 1:** Regions, SNPs, and genes reported by all types of GWAS and by DCMS

BTA	Coordinates	SNP	Genes	*P*-value	Q-value
GWAS SNPs					
15	2,551,329	BovineHD1500000589	*GRIA4*	6.97 × 10^−7^	0.0682
	2,030,345	BovineHD1500000472^a^	*MSANTD4, GRIA4*	1.26 × 10^−6^	0.0682
GWAS HTR					
15	1,991,615	ARS-BFGL-NGS-65843	*MSANTD4, GRIA4*	7.82 × 10^−7^	0.0161
	1,997,589	BovineHD1500000469			
	2,030,345	BovineHD1500000472^a^			
	2,060,428	BovineHD1500000476			
22	18,039,089	ARS-BFGL-NGS-52880	*GRM7, LMCD1*	1.29 × 10^−6^	0.0161
	18,080,658	BovineHD2200005201			
	18,127,222	BovineHD2200005211			
	18,151,738	BovineHD2200005214			
	18,158,982	BovineHD2200005216			
DCMS					
15	1,715,870	BovineHD1500000433	*MRE11*	0.000421	0.0459
	1,748,346	BovineHD1500000439	*ANKRD49; AASDHPPT*	5.74 × 10^−5^	0.0122
	1,776,278	BovineHD4100011767	*AASDHPPT*	6.42 × 10^−6^	0.0026
	1,784,872	ARS-BFGL-NGS-116439	*AASDHPPT*	4.80 × 10^−6^	0.0022
	1,798,291	BovineHD4100011768	*AASDHPPT*	4.22 × 10^−6^	0.0019
	1,838,326	BovineHD1500000457	*AASDHPPT; MSANTD4*	7.76 × 10^−6^	0.003
	1,889,648	BovineHD1500000462	*AASDHPPT; MSANTD4*	0.000111	0.0191
	1,958,234	BTA-18366-no-rs	*AASDHPPT; MSANTD4*	8.03 × 10^−5^	0.0153
	1,991,615	ARS-BFGL-NGS-65843	*MSANTD4*	6.88 × 10^−5^	0.0137
	1,997,589	BovineHD1500000469	*MSANTD4*	6.12 × 10^−5^	0.0127
	2,030,345	BovineHD1500000472^a^	*MSANTD4; GRIA4*	6.88 × 10^−5^	0.0137
	2,045,135	Hapmap52632-rs29014915	*MSANTD4; GRIA4*	0.000114	0.0194
	2,060,428	BovineHD1500000476	*MSANTD4; GRIA4*	0.000412	0.0455
	2,082,429	BovineHD1500000480	*MSANTD4; GRIA4*	0.000504	0.0508
	2,117,657	BovineHD1500000487	*GRIA4*	0.000589	0.0558
	2,121,835	Hapmap50196-BTA-105647	*GRIA4*	0.000402	0.045

^a^A common SNP reported by all types of analyses on BTA15

### Haplotype-based genome-wide association study

A total of 24,873 LD blocks were identified in our dataset ranging from 2 to 63 (with median being 3) SNPs and from two base pairs up to 1.57 Mbp (median length is 43.9 Kbp) in the cattle genome (see Additional file 1: Figure S3), covering 62% of the total length of the bovine autosomes. After filtering for low frequency haplotypes (< 1%) within the LD blocks, the number of haplotypes varied from 2 to 22 per block with the median number of 4 (see Additional file 1: Figure S4). The haplotype trend regression results demonstrated some inflation of test statistics (see Additional file 1: Figure S2) with the λ estimate being 1.17. As unlike EMMAX model, the HTR does not account for confounding effects such as population structure or family relatedness, this could be the reason for the inflation observed. To correct for the inflation, we rescaled each statistic value using a standard genomic control procedure with λ being brought to one. After this adjustment two genome-wide LD blocks reached the significance threshold (q-value < 0.05) (Fig. 1). The most significant LD block (*p*-value = 7.82 × 10^− 7^, q-value = 0.016; Fig. 2 and Table 1) was found on BTA15 and was composed of four SNPs. One of these SNPs (BovineHD1500000472) was the second top SNP in the SNP-based GWAS. Other SNPs were also found in proximity to the *GRIA4* and *MSANTD4* genes. The second significant LD block (p-value = 1.29 × 10^− 6^, q-value = 0.016; Fig. 1; Table 1) was found on BTA22. It contained five SNPs lying in the intergenic region between *GRM7* and *LMCD1* genes.

### Scans for signatures of selection results

The de-correlated composite of multiple signals (DCMS) analysis (see Additional file 3) reported 1493 SNPs distributed along 86 regions under putative selection in our complete dataset and found on 22 cattle autosomes (see Additional file 3). The sizes of these intervals ranged from 1 bp to 3.3 Mbp with a mean of 246.4 Kbp (see Additional file 3). A total of 174 genes were found within these regions. The strongest signals of putative selection (*p*-value < 1 × 10^− 10^) were detected on BTA1, 2, 5, and 14. On BTA15 we detected a 448.4 Kbp interval completely overlapping with the most significant region reported by our haplotype-based GWAS. One SNP overlap (BovineHD1500000472) was also observed between this region and the SNP GWAS suggestive region on BTA15. The DCMS interval contained five genes: *MRE11*, *ANKRD49*, *AASDHPPT*, *MSANTD4,* and *GRIA4*. In addition, DCMS reported regions containing top-ranked genes that could potentially be involved in adaptation to cold climate, including *MEF2A* (myocyte enhancer factor 2A), known to be involved in acclimation in fish [14], *NBEA*, found to be associated with support of body temperature in cattle during heat stress [10] and also known to contribute to body weight and feed intake [15]. Top-ranked genes in other DCMS regions are well known to be related to domestication and pigmentation in cattle and other species (*EDN3*; [16], carcass weight (*FAM110B, TOX*; [17]), growth (*FGF6*, *LCORL*, *XKR4*; [18]), reproduction (*PKP2*, *OPRK1*; [19, 20]), cell division and development (*WIF1;* [21]).

## Discussion

The main aim of this study was to identify chromosome intervals and genes related to the ability to maintain body temperature during exposure to acute cold winter temperatures in cattle from Siberia. A population consisting of two related beef cattle breeds has been used for this experiment. The Hereford is one of the old beef breeds established in Europe back in the XVIII century and historically is considered to be cold adaptable [22]. It has a proven record of successful breeding in the Russian Federation (Southern Ural area) where the mean winter temperature falls below -20 °C [23]. The Herefords used in our study were from herds bred in Siberia since the 1960s. The second breed, the Kazakh Whiteheaded, was established between the 1930s and 1950s in the Kazakh Republic of the USSR, by crossing Herefords with the native Turano-Mongolian Kazakh and Kalmyk cattle [24]. The Kazakh Whiteheaded is well-adapted to the continental climate of Kazakhstan. We recently confirmed a very close genetic relationship between the Kazakh Whiteheaded and Hereford breeds in our study of genetic history of Russian cattle breeds [25]. The genetic similarity of these two breeds is further confirmed by their phenotypic resemblance.

To our best knowledge, this study is the second GWAS focused on maintenance of body temperature under cold stress in cattle and it is the first one focusing on the Hereford and related breeds from Siberia. We integrated GWAS with searches for genomic selective sweeps utilizing the same testing population. Both approaches have limitations, but their integration into a single study provides an additional level of confidence to our results. The fact that the SNP-based GWAS was capable of detecting a single chromosome region only at a suggestive level of significance (FDR < 0.1) is most likely explained by a relatively small size of our testing population (~ 200 individuals) coupled with stringent thresholds applied to account for multiple testing error (> 100,000 SNP markers were used). The later issue was addressed in a second, haplotype-based GWAS with the number of independent markers (haplotype blocks) reduced to ~ 24,000. While the haplotype-based analysis points to an additional associated region on BTA22, both methods pointed to an overlapping region on BTA15 being associated with body temperature maintenance in the Siberian cattle population under acute winter cold temperatures.

It is expected that if a haplotype advantageous for a trait (e.g., cold resistance) is not fixed in the population (fixation for cold resistance traits is expected for breeds established in regions with very cold climate, e.g., the Yakut cattle [26]), it could still be under selective pressure. To test this and to provide an additional support for the associated region(s) to be related to cold resistance, we performed scans for signatures of selection (selective sweeps) for the same cattle population. The scan pointed to multiple narrow genome intervals under putative selection in our population, most of which contained genes known to be under selection in cattle including the beef cattle breeds. This confirms that selective sweeps found in our study were likely formed due to selective pressure rather than being artefacts of our analysis. In addition to the regions known to be related to economically important traits and domestication (e.g., *EDN3*, involved in coat colour traits in cattle), DCMS identified two chromosome intervals containing top-ranked genes known to be related to thermoregulation. None of these regions were detected in our GWAS, suggesting that either these genes are not involved in reaction to acute cold exposure or that the power of our GWAS was not high enough to detect weaker signals. Nonetheless, we identified a selective sweep on BTA15 which completely overlapped with the results of the haplotype-based and SNP GWAS, demonstrating that this region is likely to be under selective pressure in our population. Therefore, the combination of GWAS and scans for signatures of selection point to a single region in the cattle genome associated with body temperature maintenance under the acute cold stress in beef cattle from Siberia.

The GWAS regions contained two genes *MSANTD4* and *GRIA4,* while the selective sweep in this region was wider, covering five genes including the *MSANTD4* and *GRIA4.* This fact makes these two genes our top candidates. The *MSANTD4* is a not well-characterized gene from the Myb/SANT domain-containing gene family mainly coding for transcription factors (https://www.genenames.org/cgi-bin/genefamilies/set/532). To the best of our knowledge there is no direct connection between *MSANTD4* and thermoregulation. However, there is a link between *MSANTD4* and the heat shock protein (*HSPB1*) in humans with *MSANTD4* being a known repressor of *HSPB1* [27]. Many members of heat shock protein family are involved in response to heat and cold stresses [28] and other stresses as well [29]. Consistent with this, Mohanarao and colleagues (2014) demonstrated that *HSPB1* increases expression level in goat peripheral blood mononuclear cells in response to short-term cold stress [30].

The second candidate gene, *GRIA4*, encodes for the glutamate ionotropic receptor AMPA type subunit 4, which mediates excitatory synaptic transmission [31]. Glutamate receptors mediate most of the excitatory neurotransmission in the central nervous system of mammals, effecting plastic changes in the brain including memory, learning, and formation of neural networks during development [32]. AMPA receptors are expressed in the hypothalamus [33], which controls thermoregulation in mammals [34]. It was shown that activation of AMPA receptors in the medial preoptic area of the hypothalamus leads to a rise in body temperature in rats [35], suggesting that *GRIA4* expression could be involved in the thermoregulation response to acute cold stress in cattle as well. As a next step, resequencing of the whole candidate chromosome region as well as an RNASeq analysis of all five genes found within this region will be required to check for differences in sequence content and level of gene expression between individuals exhibiting contrasting forms of the phenotype in order to find gene(s) with genetic variants and/or level of expression differences consistent with the segregation of the phenotype.

## Conclusions

To our best knowledge, this research is the first study integrating the GWAS and detection of signatures of selection approaches in a single experiment to reveal chromosome intervals which could control body temperature maintenance in response to acute cold stress in cattle from Siberia. Our results point to a novel region on BTA15 found on an intersection of results from both methods as the best candidate region associated with the cold-resistance phenotype. The overlapping region contains two genes, the *GRIA4* and *MSANTD4,* of which *GRIA4* could be considered the best candidate based on its known function. However, it cannot be excluded that other genes in this region contribute to thermoregulation in cattle, meaning that further studies of this chromosome region including sequencing are required. The results of this study and the follow up studies could contribute to the development of cattle breeds better adapted to the extremely cold climates of the Russian Federation and other countries with cold climates.

## Methods

### Sample collection and phenotype measurements

A total of 197 animals of the Hereford and Kazakh Whiteheaded breeds aged from 6 months to 13 years were used for ear canal temperature measurements for two weeks in February 2017 using an FC-409 active RFID ear tag attached to a temperature sensor (Friendcom, China). Temperature measurements for each animal were transmitted every 15 min to a receiver connected to a personal computer. The procedure had been started a few days before the coldest period of the month as predicted from the meteorological forecast, according to recommendations from Howard and co-workers (2014) [10]. After the measurements were completed, the period of five coldest days containing the minimal temperatures (down to -32 °C) within the two-week measurement period was chosen based on temperature records from https://rp5.ru/Weather_in_Tselinnoye,_Altai_kray. Then, the area under the curve of body temperature over this five-day interval was calculated for each of the 197 animals by averaging temperature measurements for each hour and applying the trapezoid rule. The values obtained were considered as the “temperature maintenance” phenotype for each animal. To avoid genotyping and analyzing data originating from individuals that had temperature abnormalities, we followed NAWAC’s recommendations on the allowable core temperature range in cattle (National Animal Welfare Advisory Committee, New Zealand, www.mpi.govt.nz/dmsdocument/1417-dairy-cattle-animal-welfare-code-of-welfare-review-of-submissions-and-update). As a result, twelve animals were excluded from our dataset. Among them, eleven demonstrated body temperatures below the lower limit (36.5 °C) as a result of sensor being misplaced during the experiment and one had value above the upper boundary (40.5 °C). The remaining 185 animals were used for genotyping (see Additional file 2).

### DNA extraction and genotyping of single nucleotide polymorphisms (SNPs)

Blood samples (5–10 ml) from the caudal vein were collected from each animal into EDTA vacutainer tubes (Weihai Hongyu Medical Devices Co., Ltd., China). DNA from blood samples was extracted using cell lysation followed by phenol-chloroform extraction [36]. Genotyping was performed on the GeneSeek Genomic Profiler High-Density (GGP HD150K) array containing 139,376 SNP markers (Novogene, San Diego, USA). Genotypes were called using the GenomeStudio 2 software (Illumina, San Diego, USA). A pedigree (.ped) file containing the genotype calls, samples, breed identifiers and a map (.map) file containing the chromosomal location and identifier for each SNP were generated using GenomeStudio 2 and imported into the PLINK whole genome analysis toolkit [37] for further processing. Data filtering was carried out using PLINK v.1.9. First, we discarded all SNPs from sex chromosomes as well as those not assigned to any chromosome. No additional manipulations were applied to prepare the data for haplotype inference, haplotype block computation and subsequent haplotype-based GWAS. For the SNP-based GWAS analysis and signature of selection analysis, the dataset was further filtered to remove SNPs with a minor allele frequency (MAF) of < 0.05, strongly deviating from the Hardy-Weinberg equilibrium (*p*-value < 10^− 6^) and SNPs with a low call rate (in < 90% of samples) using the PLINK commands: --maf 0.05 --hwe 0.000001 --geno 0.1. We also calculated the number of heterozygous SNPs for each individual using the --het command in PLINK. Two individuals expressing abnormally high level (> 70%) of heterozygosity (due to possible contamination) were excluded. Therefore, 183 samples were further used in all analyses.

### Principal component analysis

To check the breed assignment for each sample for the two phenotypically and genetically similar breeds (Hereford and Kazakh Whiteheaded [25]) we performed a principal component analysis (PCA) of the whole dataset with PLINK as follows: using the filtered SNP set we further removed all SNPs with r^2^ > 0.7 within a sliding window of 100 SNPs (--indep-pairwise 100 5 0.7). Then, the PCA was performed using PLINK command --pca. We visualized the results of the first and second principal components (see Additional file 1: Figure S1). Samples from the two breeds formed two separate clusters with several samples being exceptions. The breed assignment for these samples was changed according to their clustering results.

### SNP-based GWAS

Single-point GWAS was performed using a variance component model implemented in EMMAX software [38]. This algorithm uses restricted maximum likelihood (REML) estimates of variances and accounts for both population stratification and relatedness between individuals by estimating the contribution of the sample structure to the phenotype. The statistical model used in the analysis was as follows:

$$ \mathbf{y}=\boldsymbol{\upmu} +\mathbf{X}\mathbf{\ss }+\boldsymbol{\upalpha} +\mathbf{e}, $$

where y represents an n × 1 vector of phenotype measurements (*n* = 183); μ is an n × 1 population mean vector; Xß is a fixed effect term, where X is an n × 3 design matrix for the fixed effects and ß is a 3 × 1 vector of regression coefficients; α is an n × 1 random effect vector accounting for polygenic effects whose covariance matrix is proportional to kinship matrix (i.e. α ~ N (0, Kσ^2^_α_), where K is the kinship matrix and σ^2^_α_ is the additive genetic variance); e is an n × 1 vector of residuals, assuming e ~ N (0, Iσ^2^_e_) where σ^2^_e_ is the residual variance and I is the identity matrix. Sex (coded as 1 or 2), breed (coded as 1 or 2) and genotype of the marker (coded as 0, 1 or 2, according to allele dosage) were considered as fixed effects. The kinship matrix was estimated using Balding-Nichols model (BN-matrix). We used the Benjamini-Hochberg FDR method to control for multiple testing error rate [39]. Q-values of 0.05 and 0.10 were considered as significant and suggestive thresholds, respectively.

### Computation of linkage disequilibrium (LD) haplotype blocks

The gametic phase of genotype data was inferred for each chromosome using the whole genotyping dataset, using fastPhase software v.1.4.8 with default parameters and the number of clusters (K) equal to 40, as estimated using the fastPhase cluster determination algorithm [40]. Then, genotypes with a known phase were partitioned into linkage disequilibrium (LD) blocks using Haploview software, v.4.2 [41]. We used the solid spine algorithm, which requires the first and last SNPs in a block to be in a strong LD (D’ ≥ 0.8) with all intermediate markers.

### Haplotype-based genome-wide association study

We performed the haplotype trend regression (HTR) analysis [42] (R package *gap*, function *htr* [43]) to test for association between LD blocks and the range of phenotype measurements in our dataset. For each LD block we composed an n × h matrix of haplotype dosage, where n is the number of animals studied (*n* = 183) and h is the number of haplotypes observed within an LD block. Only haplotypes with frequencies > 0.01 in our dataset were included in the analysis. Because the *htr* function required the design matrix consist of the haplotype dosages only we fitted the linear regression model relating the phenotype to sex and breed. The residual vector, containing the amount of variance unexplained by the effects of those predictors, was then considered as the adjusted phenotype. Our statistical model therefore when using the *htr* function was as follows:

$$ \mathbf{y}=\boldsymbol{\upmu} +\mathbf{D}\mathbf{\ss }+\mathbf{e}, $$

where y is an n × 1 vector of adjusted phenotype; μ is an n × 1 population mean vector; D is an n × h matrix of haplotype dosage and ß is an h × 1 vector of haplotype effects; e is an n × 1 vector of residuals, assuming e ~ N (0, Iσ^2^_e_). We were interested in testing the overall null hypothesis H_0_: ß_1_ = ß_2_ = _…_ = ß_h_ = 0 and used the F test to infer the overall significance of the regression. When defining the significance thresholds for HTR analysis we used the same q-value thresholds as in the SNP-based GWAS.

### Detecting signatures of selection

It has recently been demonstrated that the application of the composite measures of selection significantly improves signal to noise ratio and increases the power for the location of signals of selection, specifically in comparison to single statistics or their meta-analysis [44, 45]. We combined four genome-wide statistics including the haplotype homozygosity (H1 [46]), modified haplotype homozygosity statistics (H12 [46]), Tajima’s D index [47], and nucleotide diversity (Pi [48]) in the de-correlated composite of multiple signals (DCMS) framework [44] following the same procedure previously described in Yurchenko and co-workers (2018) [7] except for the SHAPEIT2 [49] parameter –effective-size being set to 500. DCMS combines *p*-values produced by several statistics for each locus into a single measure accounting for correlation between the statistics. The correlation matrix was calculated genome-wide and allowed the assignment of different weights to each statistic’s *p*-value depending on their genome-wide correlation. The resulting DCMS statistics were examined for normality of their distributions and then fitted to the normal distribution using the robust fitting of linear model method implemented in the *rlm* R function of the MASS package [50]. The fitted DCMS statistics were then converted into *p*-values using the *pnorm* function (lower.tail = FALSE, log.p = FALSE) and the *p*-values were finally converted to the corresponding q-values using the *qvalue* R function [51].

### Gene identification and data visualization

Genes within the associated regions and those adjacent to them were identified using the UMD_3.1.1/bosTau8 assembly of the cattle genome from the UCSC Genome Browser [52]. For the GWAS results genes were identified within ±250 Kbp from the associated SNPs. For the DCMS statistics that could potentially cover large intervals with many SNPs, we considered chromosome intervals with SNPs with adjusted *p*-values of < 0.05, and boundaries of each interval were defined by the locations of the first flanking SNPs exhibiting adjusted *p*-values of > 0.1. Within the selected intervals, genes were identified within 1σ value from the most significant SNP based on a statistical values distribution similar to Yurchenko and co-workers (2018) [7]. This approach results in fewer candidate genes being reported for the “sharp” selection peaks while for the intervals with many SNPs exhibiting similar statistics values, larger numbers of genes were reported. Genes were also ranked based on their distance from the SNP with the highest statistical value in each region with larger ranks assigned to more distant genes. Manhattan plots for all types of analyses were built with a script written on the basis of the Matplotlib Python library [53].

## Additional files

Additional file 1: **Figure S1**. Results of Principal Component Analysis (PCA) for Hereford and Kazakh Whiteheaded breeds. **Figure S2**. Q-Q plots for GWA analyses. **Figure S3**. Distribution of haploblock lengths. **Figure S4**. Number of haplotypes in haploblocks. (DOCX 4181 kb)

Additional file 2: Description of the experimental dataset: breeds, sex, phenotype. (XLSX 12 kb)

Additional file 3: Putatively selected regions of the cattle genome in the Hereford and Kazakh Whiteheaded breeds as reported by the DCMS statistics. (XLSX 16 kb)
