# Supplementary material for: Genome-wide association study and scan for signatures of selection point to candidate genes for body temperature maintenance under the cold stress in Siberian cattle populations
Source: BMC Genet. 2019 Mar 18;20(Suppl 1):26. doi: 10.1186/s12863-019-0725-0 (PMC6421640; doi:10.1186/s12863-019-0725-0)
Supplement: Supplementary file 1 — Figure S1. Results of Principal Component Analysis (PCA) for Hereford and Kazakh Whiteheaded breeds. Figure S2. Q-Q plots for GWA analyses. Figure S3. Distribution of haploblock lengths. Figure S4. Number of haplotypes in haploblocks. (DOCX 4181 kb) [file 12863_2019_725_MOESM1_ESM.docx]

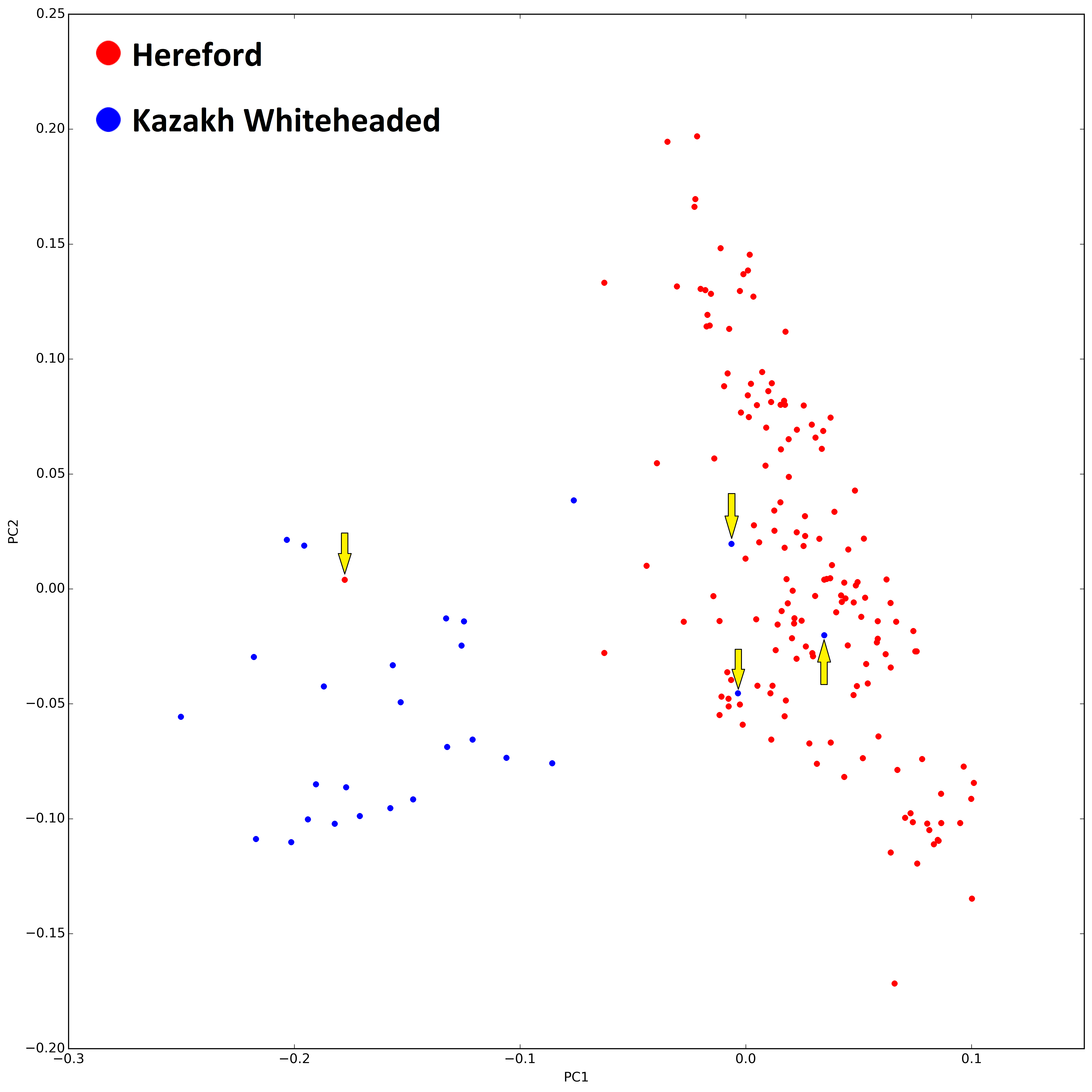


**Figure S1.** Results of Principal Component Analysis (PCA) for Hereford and Kazakh Whiteheaded breeds. Yellow arrows indicate individuals for whom breed ID were changed for our analyses.


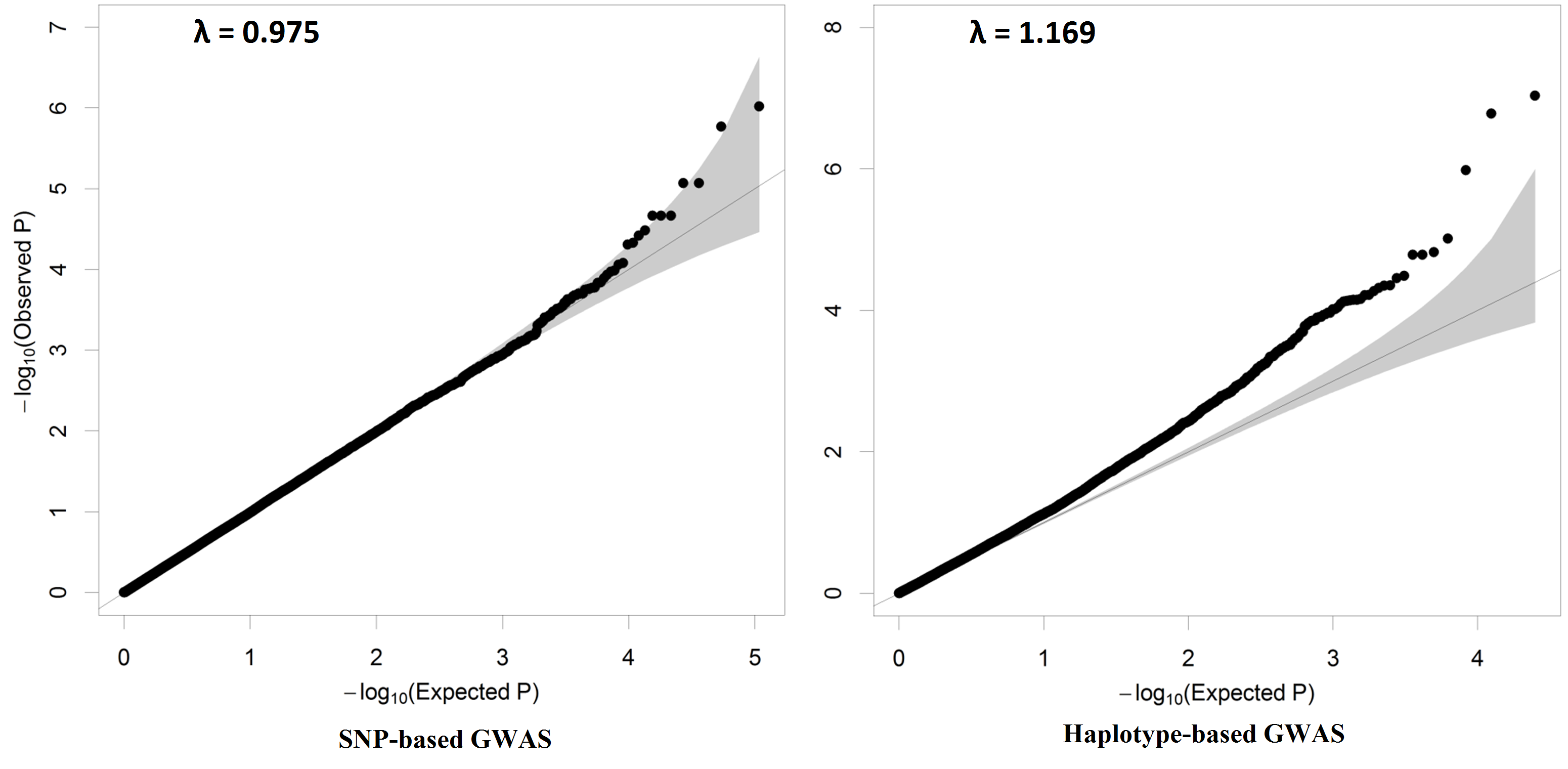


**Figure S2.** Q-Q plots for GWA analyses.


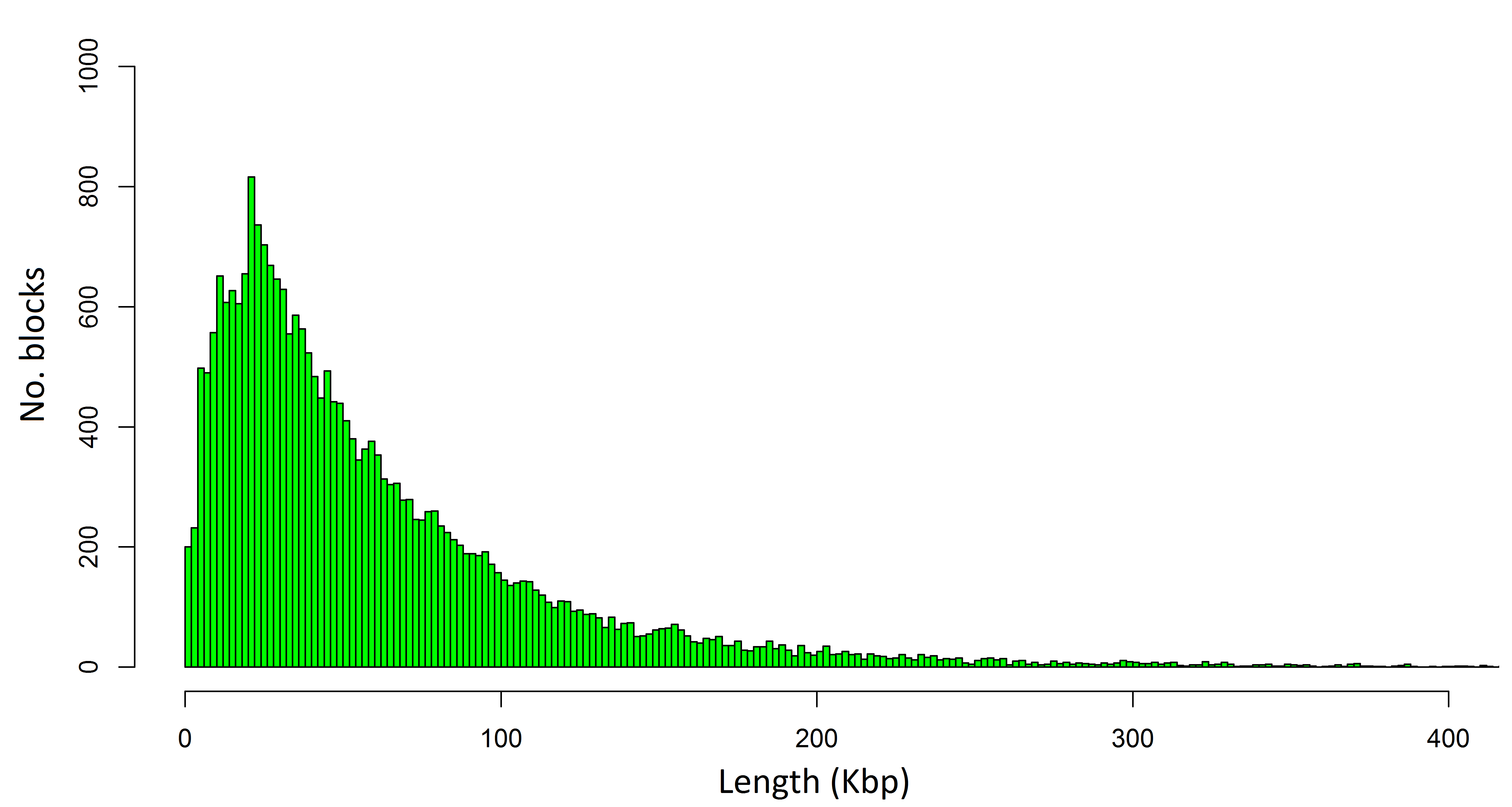


**Figure S3.** Distribution of haploblock lengths.


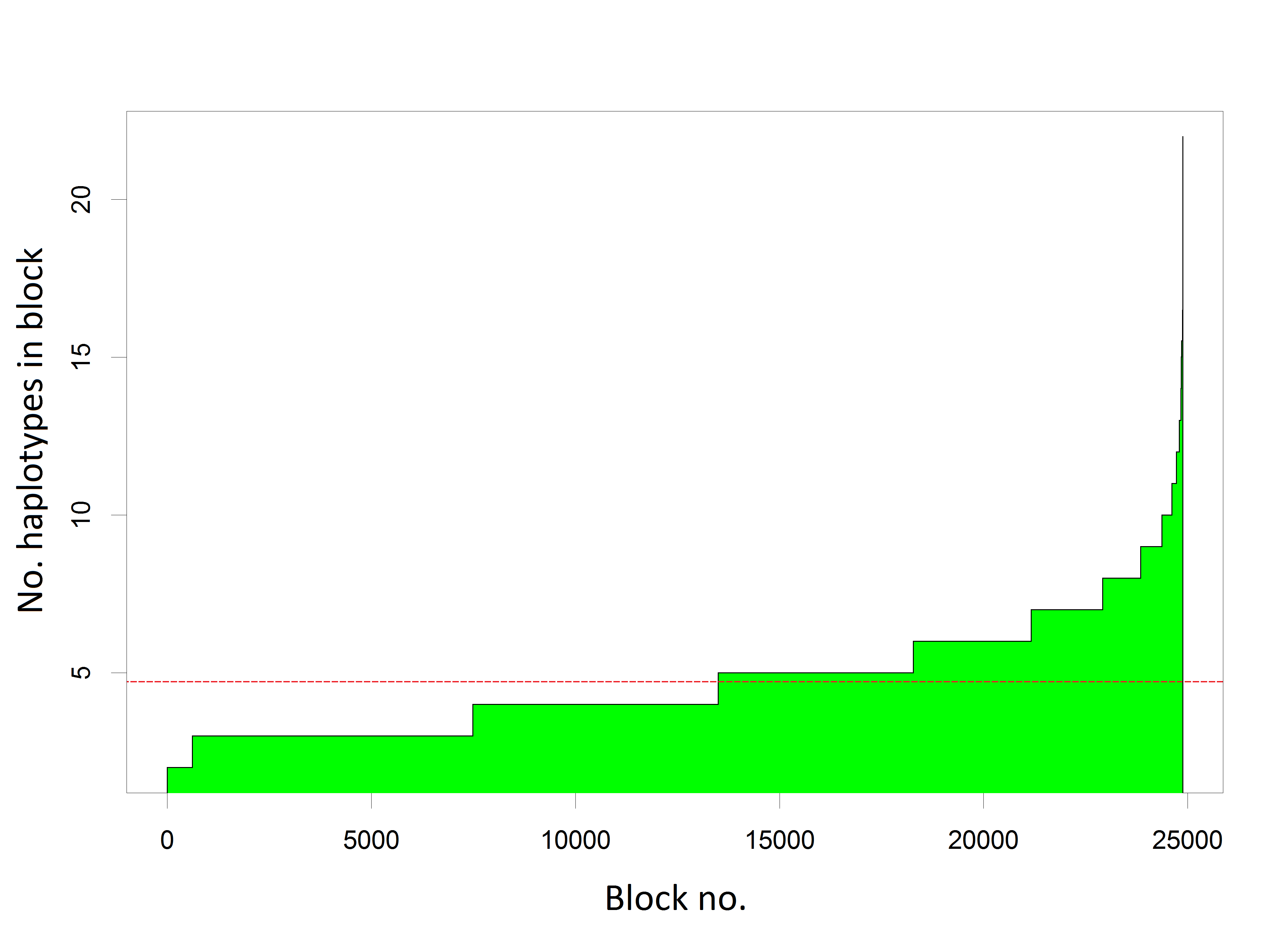


**Figure S4.** Number of haplotypes in haploblocks. The red dotted line shows the mean number of haplotypes in haploblocks. Data are shown for the filtered haplotypes (frequency >1%) in haploblocks.
